# Unique cases of large and small bowel obstruction in intraperitoneal renal transplantations: a case series and review of literature

**DOI:** 10.1093/jscr/rjad640

**Published:** 2023-11-29

**Authors:** Zirong Yu, Ferdinand Ong, Vijay Kanagarajah

**Affiliations:** Department of General Surgery at Princess Alexandra Hospital, Metro South Health in Princess Alexandra Hospital, 199 Ipswich Rd, Woolloongabba, QLD 4102, Australia; Department of General Surgery at St George Hospital, South Eastern Sydney Local Health District in St George Hospital, Gray St, Kogarah, NSW 2217, Australia; Department of General Surgery at Princess Alexandra Hospital, Metro South Health in Princess Alexandra Hospital, 199 Ipswich Rd, Woolloongabba, QLD 4102, Australia

**Keywords:** bowel obstruction, transplant, kidney, pancreas, intraperitoneal

## Abstract

Bowel obstruction is a common cause for the acute abdomen with different aetiologies that shapes subsequent management plans. Small bowel obstruction often develop due to intra-abdominal adhesions in patients with prior abdominal surgery and for large bowel obstructions, more commonly due to tumours and lesions. Disruptions to normal intra-abdominal anatomy as seen in pancreatic–kidney transplantation or kidney transplant alone can result in increased risk of bowel obstruction—especially if the donor graft is implanted within the intraperitoneal plane. We present two patients from separate institutions with history of simultaneous pancreas–kidney (SPK) transplantation (Patient 1) and intraperitoneal renal (Patient 2) transplant whom both presented with bowel obstruction requiring surgical intervention. Given the specificity and operative intricacies of our cases, we aim to present our findings and surgical management of these rare presentations in hopes of increasing awareness to this uncommon but significant cause of bowel obstruction in a transplant patient.

## Introduction

Bowel obstruction is a relatively common cause of abdominal pain and presentation to the emergency department. It is associated with a high risk of perforation and is a major cause of morbidity and mortality [1, 2]. Treatment is dependent on the cause of the obstruction [3]. We present two cases from separate institutions, one of a large bowel obstruction (LBO) related to the stented ureter of a simultaneous pancreas–kidney (SPK) transplantation and the other of a small bowel obstruction (SBO) in a transplant ureteric anastomosis to a previous ileal conduit. These cases both display rare and unusual causes of bowel obstruction and are important considerations for possible posttransplant complications.

## Case presentation—Patient 1

A 47-year-old female presented to the emergency department with severe abdominal pain, nausea, and vomiting that developed acutely overnight. She had recently been discharged from hospital 4 days earlier, following an admission for urosepsis secondary to ureteric obstruction of her renal transplant graft. In that admission, she was treated with intravenous meropenem, percutaneous nephrostomy, and stenting of her transplanted ureter.

Her background medical history included type 1 diabetes mellitus with significant micro and macrovascular complications, involving profound gastroparesis and diabetic nephropathy which she received an SPK transplant 2 years prior for end-stage renal failure. Her past surgical history is notable for extensive peripheral vascular disease resulting in a right below knee amputation as well as diabetic retinopathy with bilateral cataract surgeries. Her regular medications included triple immunosuppression with prednisolone, tacrolimus, and mycophenolate.

The patient reported feeling generally well since her recent hospital discharge, denied any pain, fevers or urinary symptoms, and tolerated substantial oral intake. However, she had not opened her bowels for 4 days. On arrival to hospital, she was hypotensive, tachypnoeic, and hypothermic with a Glasgow Coma Scale score of 11. Initial examination demonstrated cool and clammy peripheries with no palpable radial pulse. Her abdomen was grossly distended and diffusely tender on palpation. Immediate resuscitation was commenced with intravenous fluids, antibiotics, and a noradrenaline infusion was started.

**Figure 1:**
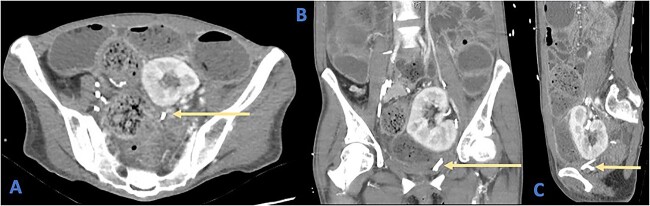
Axial (A), coronal (B), and sagittal (C) views of a CT demonstrating the position of the ureteric stent (arrows) as it crosses the rectosigmoid colon. Note the faecalization of proximal colon and dilated colonic loops.

Her full blood count and biochemical analysis were relatively unremarkable with a white cell count (WCC) of 5.2 × 10^9^/l, haemoglobin (Hb) of 100 g/l, c-reactive peptide (CRP) of 15 mg/l and arterial blood gas showing a serum lactate of 0.6 mmol/l and an acidemia with pH of 7.26. Bedside urinalysis demonstrated leucocytosis and microscopic haematuria. Computed tomography (CT) scan of her abdomen and pelvis revealed diffuse dilatation of the large bowels with a clear transition point at the rectosigmoid junction (Fig. 1). There was no evidence of viscus perforation or ischemia.

A decision was made for urgent surgical intervention due to her rapidly deteriorating haemodynamic status despite appropriate fluid resuscitation.

**Figure 2:**
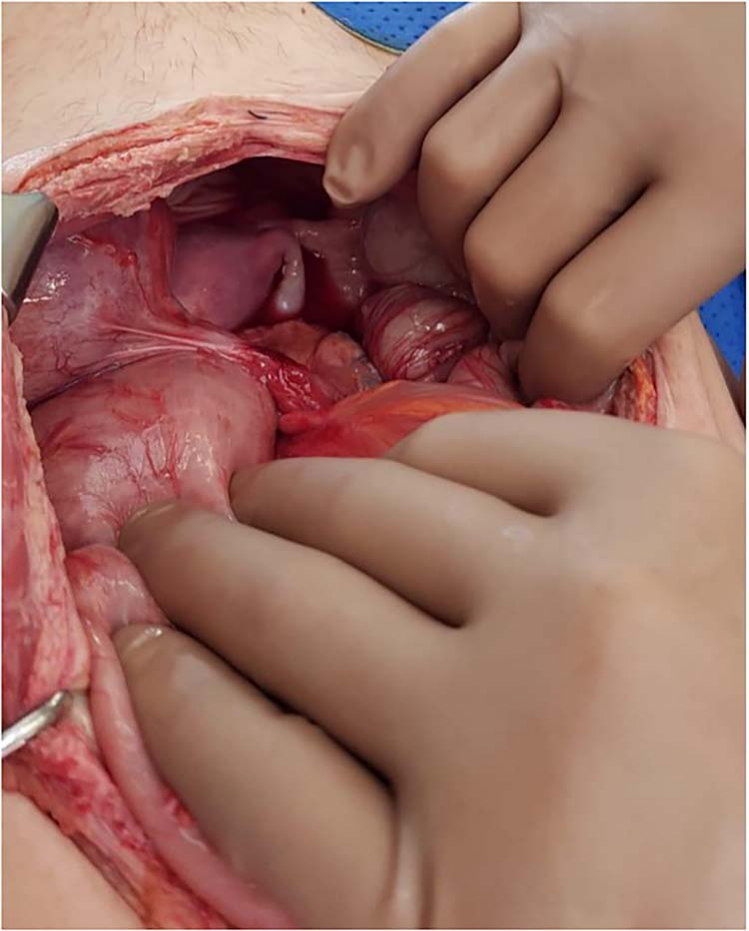
Intraoperative photo demonstrating a cord-like structure crossing over the rectosigmoid colon. This was thought to be the ureter with stent *in situ*.

The patient underwent a midline laparotomy which revealed extensive adhesions requiring significant dissection in order to adequately visualize and assess the viscera. Intraoperative examination revealed large volumes of turbid serous fluid in the abdominal cavity. Identification of the transplanted pancreas showed its enteric anastomosis with the proximal small bowel, a venous anastomosis to the right external iliac vein, and an arterial anastomosis to the right common iliac artery. The transplanted kidney was situated in the left iliac fossa and was anastomosed to the left external iliac vein and the left common iliac artery. The transplanted ureter was anastomosed to the dome of the bladder and given its anatomical placement, was situated intraperitoneally (Fig. 2).

**Figure 3:**
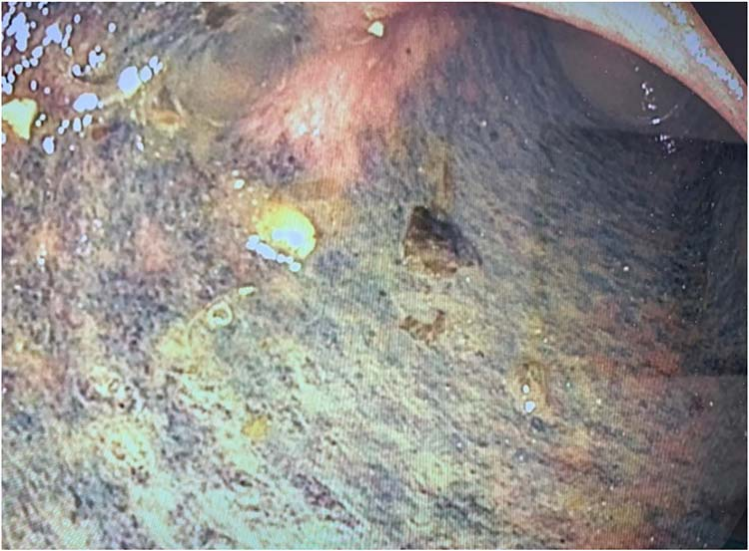
Photograph of the mucosal ischaemic changes in the part of the colon distal to the area of compression by the cord-like structure (presumably the stented ureter).

The colon was visibly dilated and macroscopically appeared viable with no external evidence of ischemia; however, on-table flexible sigmoidoscopy revealed an acute angulation at the rectosigmoid junction with evidence of mucosal ischemia distally (Fig. 3). Immediately adjacent to this area of obstruction extraluminally was the transplanted ureter with stent *in situ*. On discussion with her transplant surgeon, a de-functioning loop colostomy was created.

Postoperatively, the patient was transferred to the intensive care unit for a further 4 days before being stepped down to the ward. She recovered well and remained in hospital for another week with ongoing stoma education before being cleared for discharge home. The patient returned 6 weeks later for a planned stent exchange, which proceeded without any issues. Her colostomy was functioning normally, and she had no further symptoms since her discharge. Her ongoing follow-up was transferred back to her usual transplant surgeon and renal physician, and a joint decision was made to keep her colostomy.

## Case presentation—Patient 2

A 71-year-old male was referred in by GP for sudden onset of colicky lower abdominal pain associated with bilious vomiting and fevers. This was on background of a live right renal transplant 14 years ago for reflux nephropathy. The transplanted ureter was anastomosed to a pre-existing ileal conduit that was previously fashioned due to a congenital hypoplastic kidney in the right iliac fossa. His other medical history was notable for significant ischaemic heart disease, peripheral vascular disease, and recurrent skin cancers in context of his long-standing immunosuppression. On initial examination, his abdomen was noticeably distended and tender centrally with localized peritonism. His ileal conduit was pink and appeared healthy, however had markedly reduced urine output.

**Figure 4:**
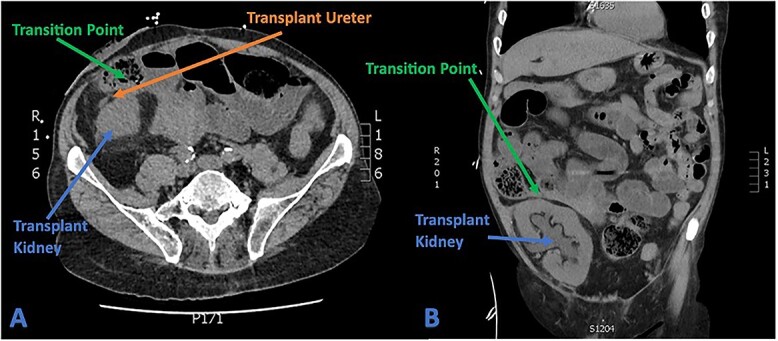
(A) Axial CT section showing transplant ureter coursing anterior to kidney transversing over the transition point of SBO. (B) Coronal CT section showing faecalized small bowel approaching transition point with gross hydroureter and hydronephrosis of transplant kidney.

His full blood count and biochemistry showed a significant leucocytosis with a WCC of 36.2 × 10^9^/l, serum lactate of 6.6 mmol/l, and severe decline in renal function as demonstrated by acute rise of serum creatinine to 285 umol/l (baseline 103 umol/l). A subsequent CT scan showed substantial hydronephrosis and hydroureter of the transplanted kidney with distended small bowel loops established in close proximity to the ureteric-ileal conduit anastomosis with an identifiable transition point (Fig. 4). Initial conservative management included copious fluid resuscitation, intravenous antibiotics, and bowel decompression via nasogastric tube. A decision, however, was made for urgent operation when repeated review showed new tachycardia, hypotension, worsening pain with increased analgesic requirements, and generalized peritonism.

The patient underwent an emergency laparotomy which revealed a transition point caused by significant adhesions of the mid-small bowel that was entrapped between the ileal conduit and its anastomosis to the stretched transplanted ureter. Intraoperative examination showed a grossly dilated ureter with marked tension at the site of small bowel adherence but fortunately no evidence of ischemia. Given the ureteric connection with the ileal conduit, the ureter was anatomically positioned intraperitoneally at the time of operation. Dilated small bowels were encountered proximally with extensive intra-abdominal adhesions but no compromised bowel that required resection.

The patient was subsequently admitted to the ICU postoperatively with ongoing vasopressor requirements augmented by intravenous hydrocortisone for stress dosing. His course was complicated by multiresistant *Escherichia coli* urosepsis, which contributed to his increased vasopressor support, and was treated with intravenous meropenem under the advice of infectious disease specialists. He was soon after stepped down from ICU and made a uneventful recovery in hospital with a resolution of infective and obstructive symptoms, and discharged on Day 7 postoperation with a creatinine of 153 umol/l, eGFR 39 ml/min, and WCC of 15.2. He was followed-up with multiple outpatient appointments with no other persisting complications and on repeat bloods 1-year postsurgery had a normalized creatinine of 99 umol/l.

## Discussion

SPK or individual kidney transplantation are widely recognized management options for patients meeting specific clinical criteria usually involving end-stage renal failure and type 1 diabetes mellitus [4–8]. Transplantation of these organs alone can be associated with many graft-specific postoperative complications such as immune rejection, delayed graft function, vascular compromise, anastomotic leak, and stenosis [9, 10]. Like any open surgery, these operations can also predispose patients to other complications such as bowel obstruction, delayed wound healing, and infection with a study reporting 26% of SPK transplant patients requiring repeat laparotomy within 3 months for postoperative complications [1, 2, 11].

In most SPK transplantations, there is preferential use of the intraperitoneal approach for graft implantation, the pancreas is usually transplanted to the right iliac fossa, and the kidney to the left iliac fossa [4, 12]. The intraperitoneal space offers more capacity and is associated with fewer peripancreatic fluid collections and wound complications [12]. Renal transplantation, however, largely utilizes the extra-peritoneal approach, allowing the placement of the graft low in the pelvis which permits trimming of the donor ureter’s length to minimize distal ischemia which may result in ureteric strictures [13]. The intraperitoneal space is more favoured in small-sized paediatric renal transplant patients [14], however has been associated with a higher incidence of gastrointestinal complications in these patients such as bowel obstruction [15].

Obstruction of the large or small bowel can be a surgical emergency and is a significant cause of morbidity and mortality accounting up to 15% of all hospital admissions for the acute abdomen [16]. The aetiology of bowel obstructions can range from intrinsic luminal obstruction, intramural pathologies, and extrinsic compression with the diagnosis made readily with comprehensive imaging modalities such as CT scans [2, 16]. Adhesions, hernias and neoplasm account for up to 90% of all SBO and alternatively 60% of all LBO are provoked by cancer, with volvulus and diverticular disease accounting for another 30% of aetiologies [16]. In the immunocompromised patient, clinical signs and laboratory assessment can be masked and at times unconvincing for underlying obstruction [17], as was reflected in the relatively normal biochemical and blood results of Patient 1. Given the alteration of normal anatomy in transplant patients, increased care and vigilance must be attended to in these individuals who presents with a bowel obstruction. This is especially true for patients with intraperitoneal graft placements, as the presence of a stented ureter or structure within this compartment can act as a focal transition point for the development of a mechanical bowel obstruction, such as the case with Patient 1. This must also be considered in an intraperitoneal kidney transplant, for Patient 2, where the aberrantly positioned ureteric anastomoses to the ileal conduit represents a very possible point for obstruction as the structures cross over the small bowels. This situation would usually have been avoided in a classically extraperitoneal renal transplant.

Bowel obstruction involving transplanted renal grafts is very rare and scarcely documented within the literature. Tovmassian *et al*. [17] reported the presence of SBO in a patient with an intraperitoneally placed renal graft secondary to a previous SPK transplantation. This case, similar to our Patient 1, revealed the intraperitoneal transplant ureters as the causative site for bowel obstruction. Two other cases [13, 18] further describe the double obstruction of the bowel and ureters secondary to the internal herniation of small bowel behind the transplanted ureter necessitating small bowel resection with revision of the uretero-cystostomy in both cases. These cases all share a major similarity among themselves, that being the intraperitoneal location of the transplanted ureter, and hence the likely increased possibility of mechanical bowel obstruction that may develop as a result of an internal hernia formed behind the structure. Given the complexity of transplant patients and their varied clinical presentation, a high index of suspicion must be recognized when managing possible complications that may arise in these patients, especially in the circumstances of intraperitoneal ureteric implantation.

## Conclusion

We report two unique cases of LBO and SBO in patients that have undergone organ transplantations. Due to the intraperitoneal location of the graft ureter post transplantation, a bowel obstruction ensued requiring surgical intervention to resolve. Both patients were followed up postoperatively and have recovered exceptionally well.

## Data Availability

The data that support the findings of this study are available from the corresponding author, ZY, upon reasonable request.
